# Ivermectin in the Management of *Demodex*-associated Blepharitis: A Comprehensive Literature Review

**DOI:** 10.1097/ICL.0000000000001299

**Published:** 2026-09-28

**Authors:** Matilde Buzzi, Pietro Sponga, Andrea Angheben, Alberto Carnicci, Fabrizio Giansanti, Rita Mencucci

**Affiliations:** Department of Neuroscience, Psychology, Pharmacology and Child Health (NEUROFARBA) (M.B., A.C., F.G., R.M.), Eye Clinic, University of Florence, Florence, Italy; Implementation Research Group (P.S.), Bernhard Nocht Institute for Tropical Medicine (BNITM), Hamburg, Germany; and Department of Infectious (A.A.), Tropical Diseases & Microbiology, IRCCS Sacro Cuore Don Calabria Hospital, Verona, Italy.

**Keywords:** *Demodex* blepharitis, Ivermectin, Ocular surface disease, Rosacea, Antiparasitic therapy

## Abstract

**Objectives:**

To review the role of *Demodex* infestation in blepharitis and summarize available evidence on the efficacy and safety of topical and oral ivermectin.

**Methods:**

This narrative review was conducted through collaboration between expert ophthalmologists and infectious diseases specialists with clinical experience in the use of ivermectin. A literature search was performed in PubMed and Embase from inception to November 2025.

**Results:**

Evidence from clinical trials, observational studies, and case series was reviewed and synthesized. Ivermectin, administered orally or topically, has been investigated as a potential treatment option for *Demodex*-associated blepharitis, either as monotherapy or in combination with other therapies. Across different treatment protocols and patient populations, ivermectin use was generally associated with reductions in *Demodex* mite density, improvement in cylindrical dandruff, ocular surface parameters, and patient-reported symptoms. Reported adverse events were infrequent and predominantly mild.

**Conclusions:**

Current evidence suggests that ivermectin may represent an effective and overall safe therapeutic option for *Demodex*-associated blepharitis. Further studies are needed to establish standardized treatment regimens and to assess long-term outcomes.

*Demodex* mites are common ectoparasites that colonize the human pilosebaceous units, with *Demodex folliculorum* and *Demodex brevis* being the two species implicated in ocular disease.^1^ Although typically present in low numbers as commensals, *Demodex* overgrowth has been increasingly recognized as a contributor to chronic anterior blepharitis, meibomian gland dysfunction (MGD), and ocular surface inflammation.^2^ *D. folliculorum* resides in lash follicles and is strongly associated with cylindrical dandruff (collarettes), a pathognomonic sign of infestation, whereas *D. brevis* may affect deeper meibomian and sebaceous glands.^3^ These collarettes are composed of undigested material, keratinized cells, eggs, dead mites, and occasionally live mites and represent a direct indicator of infestation.^4^ Clinically, *Demodex* blepharitis presents with a wide spectrum of findings, including tear film instability, MGD, eyelid margin erythema, swelling, lash misdirection, and lash loss.^5^ Patients may also experience recurrent chalazia and, in rare cases, more severe complications such as corneal vascularization, phlyctenule-like lesions, and other corneal opacities.^6^ Patients typically report symptoms such as itching, burning, eyelid irritation, and dry eye, often refractory to conventional treatments.^7^ In addition, *Demodex* mites may contribute to the pathogenesis of rosacea, a dermatological condition frequently associated with blepharitis.^8^ Overall, the disease burden of *Demodex*-associated blepharitis is considerable, with up to 80% of patients reporting a negative impact on quality of life.^9^

Despite these well-described clinical associations, the pathogenic role of *Demodex* remains debated, as its high prevalence in asymptomatic individuals suggests that colonization alone is insufficient to cause disease. Rather, *Demodex* likely operates along a spectrum from commensal to a pathogenic organism, with clinical expression depending on mite density, host susceptibility, and local microenvironmental factors. Mites may induce direct epithelial damage, follicular distension, and obstruction of pilosebaceous and meibomian glands through the accumulation of debris, supporting a potential primary role in cases of high infestation.^7^ Conversely, in predisposed conditions such as MGD, *Demodex* may act as a secondary aggravating factor. In addition, mite-associated bacteria, particularly *Bacillus oleronius*, can further amplify inflammation through antigenic stimulation.^10,11^ At the immunological level, activation of Toll-like receptor 2 (TLR-2) and downstream cytokines (e.g., IL-1β, IL-17), particularly in genetically predisposed individuals, may drive a self-perpetuating inflammatory response.^7,12^

The prevalence of *Demodex* infestation demonstrates a striking age-related trend. Epidemiological data indicate that infestation rates rise progressively with age, affecting up to 84% of individuals by 60 years and approaching 100% in those over 70.^13,14^ This highlights not only the ubiquity of *Demodex* with advancing age but also the importance of recognizing and addressing its role in ocular surface disease within an aging population. Despite its clinical relevance, *Demodex*-associated blepharitis remains underdiagnosed, partly because of nonspecific symptoms and the lack of standardized diagnostic protocols.^15^ Conventional treatments such as lubricants, antibiotics, corticosteroids, and lid hygiene often provide limited benefit in cases where *Demodex* is the primary pathogenic factor.^7^ This has led to increasing interest in antiparasitic therapies, among which ivermectin (topical and systemic) has emerged as a promising option.^16,17^ Evidence suggests that ivermectin may be an effective option for patients with *Demodex*-associated blepharitis, especially in chronic or treatment-resistant cases.^18,19^

This narrative review synthesizes current evidence on ivermectin for *Demodex*-associated blepharitis, covering its mechanisms of action, diagnostic approaches, treatment protocols, ocular surface outcomes, and safety across the available literature.

## METHODS

The present review was conducted at the Eye Clinic, Department of Neuroscience, Psychology, Pharmacology and Child Health, University of Florence, in collaboration with the Department of Infectious Diseases of the IRCCS Sacro Cuore Don Calabria of Negrar and the Bernhard Nocht Institute for Tropical Medicine. A comprehensive computerized literature search was performed in PubMed and Embase from inception to November 2025, using the keywords “blepharitis,” “blepharitides,” “Demodex,” and “ivermectin.” No language restrictions were applied. In addition, the reference lists of all retrieved articles were manually screened to identify further relevant studies. Studies were considered eligible if their primary objective was to evaluate the effects of ivermectin-based therapies in *Demodex*-associated blepharitis. Conference abstracts, letters, narrative reviews, duplicate publications, and studies lacking retrievable raw data were excluded. This review is based exclusively on previously published studies and did not involve the collection of new data from human participants or animals; therefore, ethical approval and informed consent were not required.

## RESULTS

### Diagnosis and Evaluation of *Demodex*-associated Blepharitis

Diagnosing *Demodex*-associated blepharitis requires the integration of clinical signs with confirmatory microscopic or slit-lamp evidence. The hallmark clinical feature is the presence of collarettes at the base of the eyelashes, which are pathognomonic for *D. folliculorum* infestation (Fig. 1).^20,21^ Telangiectasia, lash misdirection, and lid margin swelling are often observed as secondary clinical criteria.^21^ Patients may present with chronic ocular irritation, itching, foreign body sensation, or redness, symptoms that often persist despite conventional treatment for dry eye or anterior blepharitis.^22,23^

**FIG. 1.**
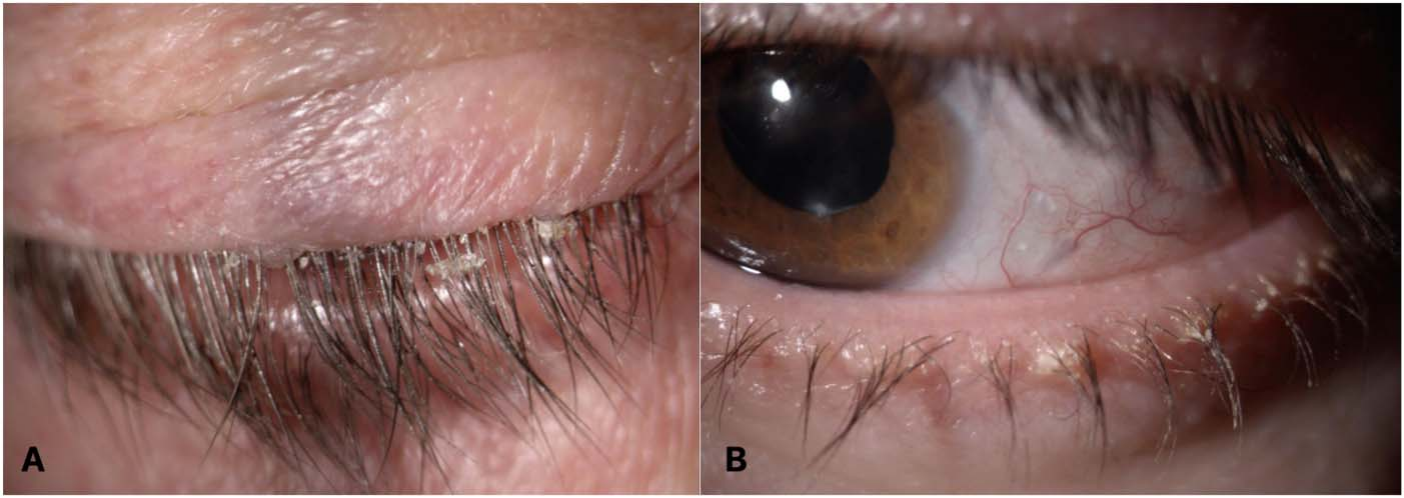
Anterior segment photographs of cylindrical dandruff (collarettes) at the base of the superior (A) and inferior (B) eyelashes, pathognomonic for *Demodex* infestation.

For confirmation of clinical suspicion, several diagnostic strategies have been used across the reviewed studies. The most common method involves epilation of eyelashes followed by light microscopy, using standard magnification ranging from ×40 to ×600. The number of lashes removed varies between protocols, from two to four lashes per lid^19,24,25^ to more comprehensive techniques removing up to 12 lashes per eye, with sampling from different lid regions.^26^ In many cases, lashes with visible collarettes or suspected mite tails are preferentially sampled.^23,27^ Microscopy-based quantification of mite density represents the diagnostic gold standard. Some authors define infestation as the presence of at least one mite per lash, whereas others require higher thresholds such as ≥3 mites per eyelid or greater than 5 mites/cm^2^ for facial lesions.^24,28,29^ Advanced approaches, including infrared meibography and digital slit-lamp documentation, have also been used to demonstrate gland dropout or mite tail extrusion.^26,30^ Some authors have proposed grading collarette severity from 0 to four based on the number of affected lashes per eye, offering a practical tool for clinical monitoring.^28^

Despite variation in methodology, the consensus across studies emphasizes the value of collarette visualization and lash sampling as key components of diagnosis. Incorporating these techniques into standard lid examination protocols may improve identification and treatment of *Demodex* blepharitis, particularly in patients with chronic, treatment-resistant symptoms.

### Ivermectin: Mechanism of Action and Clinical Applications

Ivermectin is a macrocyclic lactone with potent antiparasitic activity. It selectively binds to glutamate-gated chloride channels in invertebrate nerve and muscle cells, increasing membrane permeability to chloride ions, and leading to neuromuscular paralysis and death of the parasite.^31^ It is available in both oral and topical formulations and has been extensively studied as a versatile antiparasitic agent with established efficacy against helminthic infections, ectoparasitic infestations, dermatological conditions, and more recently as a vector control strategy in malaria-endemic regions.

In the treatment of *Onchocerca volvulus*, transmitted by *Simulium* midges and responsible for river blindness in Africa and South America, ivermectin is considered the cornerstone therapy, usually in combination with doxycycline in areas of persistent transmission.^32^ Ivermectin exhibits potent microfilaricidal activity, reducing parasitic load and alleviating complications.^33^ However, it cannot be used in areas co-endemic for *Loa loa* because of the risk of severe, potentially fatal Mazzotti reactions and encephalopathy in patients with high *Loa loa* microfilarial loads.

Ivermectin is also used as first-line therapy against *Strongyloides stercoralis*, a soil-transmitted nematode capable of chronic infection through autoinfection. Although often asymptomatic or mildly symptomatic, in immunosuppressed individuals it may progress to hyperinfection syndrome with multiorgan dissemination and high mortality rates. For this reason, screening and preventive treatment are advised before immunosuppressive therapy.^34,35^ In uncomplicated infections, a single oral dose of ivermectin (200 µg/kg) is usually effective. In hyperinfection, the optimal regimen is undefined, but ivermectin is typically administered for several days, often combined with albendazole, until clearance of larvae from biological fluids.^34,35^

In mass drug administration programs, ivermectin is used to target lymphatic filariasis, usually in combination with albendazole and/or diethylcarbamazine, aiming to reduce transmission and eliminate the disease in endemic regions.^36^

Beyond helminthic infections, ivermectin is effective against ectoparasites. It is an established alternative to permethrin for scabies, with a Cochrane review showing no significant difference in cure rates at 2 weeks, although ivermectin may act more slowly, has reduced mite clearance at 1 week, and lacks ovicidal activity, often requiring repeat doses.^37^ Oral and topical ivermectin are also effective for *Pediculus humanus capitis* (head lice), especially in treatment-resistant cases, and are considered safe alternatives to standard pediculicides.^38^

A novel application is its use as an endectocide for malaria vector control, where human administration of ivermectin results in lethal blood meals for *Anopheles* mosquitoes, reducing survival and transmission. A review of 15 studies suggested a potential role for ivermectin in malaria elimination programs, particularly in India.^39^

### Ivermectin-based Treatment Strategies for *Demodex*-associated Blepharitis

Recent literature indicates that systemic and topical ivermectin exert promising anti-*Demodex* effects, either alone or in combination with agents such as metronidazole, tea tree oil (TTO), or lubricants. Administration route, dosing frequency, and treatment duration vary widely across studies, reflecting differences in disease severity, target sites, and clinical context. Table 1 summarizes the available evidence on the use of ivermectin for the treatment of *Demodex*-associated blepharitis.

**TABLE 1.** Summary of Clinical Studies Evaluating Oral and Topical Ivermectin for the Treatment of *Demodex*-associated Blepharitis

Author (Year)	Study Type	Sample Size (Patients)	Age (years)	Treatment Regimen	Follow-up Duration
Alver (2025)	Prospective interventional study	35	Mean 49.8±15.1 years (range 18–75)	Oral ivermectin (0.2 mg/kg, two doses 30 days apart)	2 months
Ávila (2021)	Randomized clinical trial	60	Mean 55.4±19.1	Topical ivermectin 0.1% + metronidazole 1% gel	1 month
Ávila (2025)	Prospective study	54	Mean 52.3±14.1	Topical ivermectin 0.3% + metronidazole 0.5% gel	2 months
Choi (2021)	Retrospective case–control	102	Mean 63.6±12.8	Topical ivermectin 1% + hygiene	Mean 15.1±9.7 weeks
Helm (2022)	Case report	5	Not reported	Topical ivermectin 1%	1 month
Hirsch-Hoffmann (2015)	Retrospective review	32	Not reported	Oral ivermectin (6 mg on days 1 and 14)	2 months
Holzchuh (2011)	Case series	12	Mean 50.4±21.0	Oral ivermectin (200 μg/kg, two doses 1 week apart)	28 days
Huang (2024)	Prospective cohort	89	Mean 66.0±14.2 (low *Demodex* load), 69.1±11.7 (high load)	Topical daily ivermectin + tea tree oil eyelid cleansing	3 months
Nogueira Filho (2011)	Observational cohort	19	Mean 65.8 (range 48.8–82.7)	Oral ivermectin (two doses of 6 mg twice daily, 2 weeks apart)	3 months
Patel (2020)	Retrospective case series	14	Median 27 (range 11–39)	Oral ivermectin (200 μg/kg, two doses 1 week apart) + 50% tea tree oil lid scrubs	3 months
Rivera Salazar (2024)	Case series	38	Mean 61.3±15.4	Topical ivermectin 1% once monthly for 3 months	4 months
Safir (2024)	Case series	61	Mean 59.6±17.6	Topical ivermectin 1% twice daily + intense pulsed light	3 months
Salem (2013)	Randomized controlled trial	60 ivermectin alone+60 combination	Mean 36.7±12.4	Oral ivermectin alone (200 μg/kg, two doses 1 week apart) or combination of metronidazole (250 mg three times daily for 2 weeks) + ivermectin (200 μg/kg, two doses 1 week apart)	1 month
Smith (2024)	Retrospective clinical audit	75	Mean 66.6±13.9	Topical ivermectin 1% + lid hygiene	12 months
Sobolewska (2021)	Retrospective pilot study	10	Mean 56.8 (range 26–75)	Topical ivermectin 1% cream	8 months
Valvecchia (2024)	Retrospective case series	2157	Mean 50.4±15.3	Topical ivermectin 1% daily	Mean 26.1±8.5 months

#### Topical Ivermectin

Most of the reviewed studies used topical ivermectin 1% cream, typically applied to the eyelid margins and periocular skin. Regimens ranged from once-daily application (usually at bedtime) for 4 to 12 weeks^21^ to once-weekly protocols lasting several months^22,30^ or once a month for 3 months.^24^ Application techniques often involved using a cotton-tipped applicator to carefully coat the lash base and lid margin, while avoiding contact with the ocular surface. Some studies included professional in-office instillation under topical anesthesia, followed by cream removal after 10 min.^24,30^ In a recent prospective study, a daily course of topical ivermectin cream was prescribed to study participants with prominent facial symptoms or with higher facial *Demodex* densities. Facial treatment was continued for 1 to 3 months, until the resolution of facial symptoms and signs.^25^

#### Oral Ivermectin

Oral ivermectin was administered in doses ranging from single dose of 200 μg/kg to 6 mg twice daily, repeated after 14 days, most often in patients with facial demodicosis, severe blepharitis, or rosacea-associated symptoms.^25,40–42^ In one case series, two doses of oral ivermectin 1 week apart, combined with lid hygiene, resulted in substantial clinical improvement in chronic refractory blepharitis.^23^

#### Combination Therapy

Some studies have explored combined regimens of ivermectin with other anti-infective agents such as metronidazole, administered either topically or orally.^18,19,26^ This combination is supported by complementary mechanisms, with ivermectin exerting direct antiparasitic activity against *Demodex* mites and metronidazole providing antimicrobial and anti-inflammatory effects. In line with this rationale, a randomized trial comparing a topical ivermectin–metronidazole gel with placebo demonstrated a greater reduction in mite counts, particularly in patients with rosacea and anterior blepharitis.^18^ Other adjunct measures included TTO-based lid cleansers, warm compresses, intense pulsed light, and artificial tears, particularly in patients with MGD or ocular rosacea.^25,30^ Several studies emphasized the importance of eyelid hygiene continuation beyond ivermectin use, with regimens ranging from twice-daily to once-daily.^25,30^ Concomitant use of topical steroids was only permitted in selected cases for a limited duration to manage inflammation.^23^

### Clinical Outcomes and Treatment Efficacy

#### *Demodex* Eradication

In vitro, ivermectin 1% demonstrated rapid acaricidal activity against *Demodex* folliculorum by inducing irreversible cessation of mite motility within minutes of exposure, consistent with a direct neurotoxic effect on parasite neuromuscular transmission.^25,43^ In vivo, multiple studies reported how ivermectin significantly reduces *Demodex* mite density, achieving near complete or complete eradication, with both topical and systemic administration, alone or in combination. In a prospective cohort study, 76% of patients with facial *Demodex* overgrowth who received adjunctive facial topical ivermectin achieved ocular *Demodex* eradication, compared with 16% in those managed without ivermectin.^25^ In another study, microscopy-confirmed mite count reduction was observed as early as day 15 and sustained through day 30, with complete eradication reported in 71.6% of patients receiving systemic ivermectin–metronidazole combination therapy.^19^

Ivermectin was also effective in reducing collarette severity, a key clinical sign of infestation. Grading scores improved from 3.4±0.7 to 0.1±0.3 (*P*<0.01) in one cohort,^28^ and similar trends were observed for visible *Demodex* tails and lash follicle pouting.^21^ Rivera Salazar et al^24^ reported a significant reduction in cylindrical sleeves, with no sleeves observed at the fourth follow-up visit. However, some studies noted partial or delayed eradication, with one reporting a 6% full eradication rate in a nonselected patient group.^20^

#### Ocular Surface Changes

Ocular surface parameters including tear film stability, corneal staining, and subjective symptom scores were variably affected by ivermectin therapy. Tear film stability, as assessed by tear break-up time (TBUT) or noninvasive TBUT (NIBUT), improved significantly in studies using oral or topical ivermectin or combination therapy.^24,26,29,41^ However, other studies reported no significant change in TBUT.^25,28,40^

Schirmer test results were less consistent. Although one study observed a slight, nonsignificant increase in the test scores,^24^ another reported significant improvement after oral ivermectin treatment.^41^ Corneal fluorescein staining, a marker of ocular surface epithelial integrity, improved markedly in several reports.^21,22,27,29^

Across studies that reported these outcomes, MGD measures improved after ivermectin-based therapy.^26,27,42^ A significant improvement in gland secretion was reported after an intense pulsed light and topical ivermectin regimen.^27^ Also, a significantly reduced gland dropout on infrared meibography was found after ivermectin 0.3% and metronidazole 0.5% gel therapy.^26^

For conjunctival redness, a randomized clinical trial reported a marked reduction in bulbar conjunctival hyperemia/redness scores in the ivermectin–metronidazole group but not in controls,^18^ a large retrospective series found a significant decrease in conjunctival redness after topical ivermectin 1%,^28^ and a pilot rosacea cohort showed conjunctival redness improved to grade 1 in all patients after ivermectin 1% cream treatment.^29^

#### Symptom Improvement

Symptom relief was a consistent finding, with clinical improvement reported in 70% to 100% of patients across studies.^19,23–25,40–42^ Across studies, the Ocular Surface Disease Index scores consistently improved after ivermectin-based treatment, supporting a reduction in subjective ocular discomfort. Significant decreases in Ocular Surface Disease Index were reported after topical ivermectin–metronidazole therapy in randomized and retrospective studies^18,26^ and after topical ivermectin 1% in large real-world cohorts.^21,28^ Similar improvements were also observed after oral ivermectin in refractory cases.^42^ Conversely, some authors found no significant difference between treatment groups.^25,40^

Patient satisfaction was generally high after ivermectin-based therapy, with greater satisfaction reported in patients achieving clinical resolution of *Demodex* blepharitis and only a small minority expressing dissatisfaction.^27^

#### Facial Demodicosis Outcomes

Facial demodicosis outcomes improved with ivermectin-based therapy, with significant reductions in facial *Demodex* density and clinical rosacea features reported after topical ivermectin treatment.^22,25,29^ In patients with comorbid ocular and facial demodicosis, adjunctive facial ivermectin enhanced ocular *Demodex* eradication compared with nonivermectin regimens.^25^ Long-term facial improvement and sustained remission of cutaneous signs were also observed in rosacea cohorts treated with ivermectin 1% cream.^29^

### Adverse Reactions and Safety Profile

Ivermectin is generally regarded as a well-tolerated agent with a wide therapeutic window. Most adverse events are associated with *systemic* administration in infectious diseases. A 2020 systematic review of six studies, mainly from African countries, confirmed the safety of systemic ivermectin even at doses up to four times higher than standard regimens (800 vs. 200 µg/kg). Only a slight increase in transient ocular symptoms, including blurred vision, itching, ocular pain, and dyschromatopsia, was observed; all were mild or moderate, whereas severe events were rare.^44^

Neurological reactions such as vertigo and headache are uncommon, but more likely in *Loa loa* coinfection. Mutations in the *mdr-1* gene may increase susceptibility by enhancing drug penetration across the blood–brain barrier, and concomitant use of CYP3A4-interacting drugs may further contribute.^45^ In *Loa loa* infection, the principal adverse event is a potentially fatal encephalopathy, particularly in patients with high microfilarial loads. This condition is thought to result from ivermectin-induced death of microfilariae within the central nervous system, facilitated by increased blood–brain barrier permeability. Impaired consciousness typically appears 3 to 4 days after treatment, and conjunctival hemorrhages have been noted in severe cases. No consensus exists on optimal management.

Cutaneous adverse events are usually mild, most often rash and itching.^46^ Rare but severe cutaneous reactions have been reported, including Stevens–Johnson syndrome, toxic epidermal necrolysis, and DRESS, with 25 cases identified in a pharmacovigilance analysis of 517 reports, showing 20% mortality.^47^ The Mazzotti reaction, first described with diethylcarbamazine,^48^ is a well-known systemic response to ivermectin in filarial infections. It manifests with fever, tachycardia, hypotension, pruritus, pain, and lymphoedema, triggered by the release of helminthic antigens and, in onchocerciasis, by high loads of *Wolbachia* endosymbionts. Although typical of filarial disease, isolated cases suggest that it may also occur in other settings.^49^

For *Demodex*-associated blepharitis, ivermectin demonstrates a consistently favorable safety profile, with adverse events reported as rare, mild, and transient. Across published studies, no serious drug-related complications were identified, and no treatment discontinuations because of adverse events were reported.^19,21,22,24,26^ In clinical studies using topical ivermectin 1% cream, only minor local reactions such as transient skin irritation or stinging were occasionally observed; for example, a retrospective clinical audit reported mild discomfort in 2.7% of patients,^21^ whereas a large case series documented discomfort in 0.6%, none requiring discontinuation.^28^ Oral ivermectin was likewise well tolerated, with multiple studies using 200 μg/kg or 6 mg regimens reporting no systemic adverse effects, including when combined with metronidazole.^19,20,40,42^ Several investigations explicitly stated that no adverse events occurred during follow-up,^27,30,41^ whereas others did not report adverse-event data, likely because of retrospective design or limited observation periods. Ivermectin should not be used during pregnancy because safety in pregnancy has not been established.^16^

Taken together, the absence of serious or long-term adverse reactions supports the safety of ivermectin therapy, a conclusion further supported by a recent systematic review and meta-analysis reporting only mild, localized adverse events and no systemic toxicity.^16^

## DISCUSSION

Ivermectin is a long-standing antiparasitic agent with an established safety record in infectious medicine and neglected tropical diseases. Its activity against mites has led to growing interest in its use for *Demodex*-associated blepharitis. Evidence from available studies suggests that both topical and systemic ivermectin can reduce mite density and improve clinical signs such as collarettes, lash abnormalities, and eyelid margin inflammation. Some reports also describe favorable changes in tear film stability and corneal staining, with associated symptom relief in many patients. However, caution is warranted when interpreting some of the results, for example, reported improvements in meibomian gland morphology. Gland dropout is generally considered a chronic and largely irreversible change, making short-term reversal unlikely. Apparent improvements may instead reflect functional recovery or variability in meibography acquisition and grading, rather than true structural regeneration.

Safety findings for ivermectin are reassuring, with adverse events generally mild and transient, and no discontinuations reported. However, some aspects of safety remain insufficiently characterized. There are currently no adequate studies in breastfeeding women to determine infant risk or to fully characterize the concentration of ivermectin in breast milk. Ogbuokiri et al^50^ evaluated four healthy mothers and reported that ivermectin concentrations in breast milk reached approximately one tenth of the recommended safety threshold. Similar findings were reported by Rodari et al^51^ in a single patient treated for strongyloidiasis. Despite these limited data, French guidelines, based on expert consensus, suggest that ivermectin may be considered a first-line treatment for scabies in breastfeeding women when clinically indicated.^52^ Although these data derive primarily from systemic administration, they suggest that systemic exposure during breastfeeding is limited; however, specific pharmacokinetic data for topical periocular ivermectin and its ocular surface penetration remain lacking, and caution is, therefore, warranted when extrapolating these findings to ophthalmic use.

Beyond ocular involvement, ivermectin may also address concomitant facial *Demodex* overgrowth.^17^ This is particularly relevant given the proposed role of *Demodex* mites in the pathogenesis of rosacea, a dermatological condition frequently associated with blepharitis. A recent review by Wei et al^8^ supports the hypothesis that a pathophysiological continuum links erythematotelangiectatic rosacea, increased *Demodex* burden, and papulopustular rosacea, in a self-perpetuating cycle where rosacea favors *Demodex* proliferation, and mite overgrowth, in turn, exacerbates rosacea symptoms. In such cases, systemic ivermectin or topical treatment extended to facial skin may provide therapeutic benefit.^17^

During long years, lid hygiene has been used to treat *Demodex*-associated blepharitis, sometimes with sulfuric ointment, yellow mercuric ointment, pilocarpine gel, or local antibiotics without proof of efficacy.^16^ Anthelmintic agents have been used empirically in recent years, although concerns regarding systemic adverse effects remain.^16^ Other local therapy based on TTO and terpinen-4-ol have also been tested, opening new therapeutic fields.^16^ Recently, lotilaner ophthalmic solution 0.25% has been approved by the US Food and Drug Administration following the SATURN clinical trials and has been proposed as a first-line treatment for *Demodex*-associated blepharitis.^53–60^ However, it is not yet approved or commercially available outside the United States. Also, as a topical ophthalmic drop, its efficacy may be limited to eyelashes and lid margins, without penetration into periocular or facial skin. In patients with concurrent cutaneous involvement, topical ivermectin applied to periocular skin may offer broader coverage, whereas oral ivermectin remains a rational option in cases involving *D. brevis*, which inhabits deeper structures such as the meibomian glands. Oral therapy may also be considered in extensive disease affecting the face, scalp, or nasal skin, and in pediatric or adolescent patients when topical application is impractical.

The current body of literature on the use of ivermectin for *Demodex*-associated blepharitis is limited by several methodological and clinical factors. Most available studies are retrospective or observational, with relatively small sample sizes and only a few randomized controlled trials to date. This restricts statistical power and reduces generalizability. In addition, the possibility of publication bias should be considered, as the predominance of small observational studies and the relative scarcity of negative or null findings may lead to an overestimation of treatment efficacy. Considerable heterogeneity exists in diagnostic methods, including variable thresholds for mite density, inconsistent sampling techniques, and differing definitions of infestation, which complicates cross-study comparison. Similarly, treatment protocols vary widely in formulation, dose, frequency, adjunct therapies, and duration of treatment. Reported eradication rates vary substantially (approximately 6% to >70%), and this variability is likely influenced by differences in patient selection, baseline mite burden, and clinical phenotype. In particular, the presence of concomitant facial demodicosis, coexisting rosacea, and the severity of MGD may act as key confounders affecting treatment response, yet these factors are inconsistently reported across studies. This limits comparability and underscores the need for more stratified analyses or standardized reporting frameworks in future research. Direct comparisons between topical and systemic ivermectin, and between monotherapy and combination strategies, are notably lacking, and long-term follow-up and recurrence rates remain insufficiently studied. The interpretation of “eradication” should be approached with caution, as recurrence rates are poorly reported and the short life cycle of *Demodex* (approximately 14–18 days) may predispose to reinfestation in the absence of long-term follow-up data. Although continued safety monitoring is advisable for long-term or repeated use, existing data provide strong reassurance for clinical practice.

In conclusion, *Demodex*-associated blepharitis is an underrecognized yet clinically significant condition. Current evidence suggests that ivermectin, administered topically or systemically, may be an effective and generally well-tolerated therapeutic option, particularly for refractory, diffuse, or severe cases. However, the available evidence remains limited and is largely derived from small, heterogeneous, and predominantly nonrandomized studies. Future research should focus on high-quality randomized controlled trials comparing ivermectin to standard care and emerging agents. Further studies are also needed to define optimal dosing regimens, treatment duration, and long-term outcomes, including recurrence rates. Establishing consensus diagnostic guidelines and optimizing treatment duration, frequency, and application techniques will be key to sustaining long-term efficacy.
